# Friendship interventions for children with neurodevelopmental needs: A systematic review and meta-analysis

**DOI:** 10.1371/journal.pone.0295917

**Published:** 2023-12-14

**Authors:** Reinie Cordier, Lauren Parsons, Sarah Wilkes-Gillan, Matthew Cook, Matthew McCloskey-Martinez, Pamela Graham, David Littlefair, Cally Kent, Renée Speyer

**Affiliations:** 1 Department of Social Work, Education and Community Wellbeing, Northumbria University, Newcastle upon Tyne, United Kingdom; 2 Faculty of Health Sciences, Curtin School of Allied Health, Curtin University, Perth, Australia; 3 Faculty of Health Sciences, Department of Health & Rehabilitation Sciences, University of Cape Town, University of Cape Town, Cape Town, South Africa; 4 Faculty of Medicine and Health, Sydney School of Health Sciences, The University of Sydney, Sydney, NSW, Australia; 5 Department Special Needs Education, University of Oslo, Oslo, Norway; 6 Department of Otorhinolaryngology and Head and Neck Surgery, Leiden University Medical Centre, Leiden, The Netherlands; Cinvestav-Merida, MEXICO

## Abstract

**Rationale:**

Children with neurodevelopmental disorders such as attention-deficit hyperactivity disorder (ADHD), autism, developmental language disorder (DLD), intellectual disability (ID), and social (pragmatic) communication disorder (SPCD) experience difficulties with social functioning due to differences in their social, emotional and cognitive skills. Previous systematic reviews have focussed on specific aspects of social functioning rather than broader peer functioning and friendships.

**Objective:**

To systematically review and methodologically appraise the quality and effectiveness of existing intervention studies that measured friendship outcomes for children with ADHD, autism, DLD, ID, and SPCD.

**Method:**

Following PRISMA guidelines, we searched five electronic databases: CINAHL, Embase, Eric, PsycINFO, and PubMed. Two independent researchers screened all abstracts and disagreements were discussed with a third researcher to reach consensus. The methodological quality of studies was assessed using the Cochrane Risk of Bias Tool for Randomised Trials.

**Results:**

Twelve studies involving 15 interventions were included. Studies included 683 children with a neurodevelopmental disorder and 190 typically-developing children and diagnosed with either autism or ADHD. Within-group meta-analysis showed that the pooled intervention effects for friendship across all interventions were small to moderate (z = 2.761, p = 0.006, g = 0.485). The pooled intervention effect between intervention and comparison groups was not significant (z = 1.206, p = 0.400, g = 0.215).

**Conclusion:**

Findings provide evidence that some individual interventions are effective in improving social functioning and fostering more meaningful friendships between children with neurodevelopmental disorders and their peers. Effective interventions involved educators, targeted child characteristics known to moderate peer functioning, actively involved peers, and incorporated techniques to facilitate positive peer perceptions and strategies to support peers. Future research should evaluate the effectiveness of friendship interventions for children with DLD, ID and SPCD, more comprehensively assess peer functioning, include child self-report measures of friendship, and longitudinally evaluate downstream effects on friendship.

## Introduction

Several neurodevelopmental disorders have been associated with social functioning difficulties during childhood Becker, Luebbe [1]. Of interest to this study are children with neurodevelopmental disorders where social functioning is impacted due to differences in the social, emotional, and cognitive skills that are needed for successful, positive interactions with peers. This study focused on children with a diagnosis of attention-deficit hyperactivity disorder (ADHD), autism, developmental language disorder (DLD), intellectual disability (ID), and social (pragmatic) communication disorder (SPCD), as they represent prevalent neurodevelopmental disorders that impact on children’s social functioning [2,3].

ADHD and DLD are two of the most common disabilities affecting children today. Globally, an estimated 5.9% to 7.1% of children and young people have been diagnosed with ADHD, and a recent population-based study found that 7.6% of 4 to 5-year-old children presented with DLD [4,5]. Many studies have also identified strong associations between ADHD and language impairments, with comorbidity rates estimated to be between 3 and 5% [6]. While global autism prevalence estimates are much lower than those of ADHD and DLD, ranging from 0.01% to 4.4% [7], individuals on the autism spectrum often access services at much higher rates. For example, 33% of the participants in Australia’s National Disability Insurance Scheme have a primary diagnosis of autism, making it the largest diagnostic group accessing the scheme for interventions and support for participation and functioning (National Disability Insurance) [8]. Comorbidities among ADHD and autism are also common. For example, 14% of children with an ADHD diagnosis in the USA have also been diagnosed with autism. Global prevalence data estimates 1.8% of children have been diagnosed with an intellectual disability [9], with high rates of co-occurring autism, ADHD, and delayed language symptoms for children with intellectual disability [4,10]. The prevalence of SPCD is not well established, as the boundaries between autism, SPCD and a core language disorder are unclear, and differential diagnosis is hindered by a lack of suitable assessment tools [11,12]. Pragmatic language problems have been identified in 7.5% of children at school entry age. However, this is not a pure indication of SPCD as some children most likely fall within the borders of other diagnostic groups that exhibit pragmatic language difficulties (i.e., autism or ADHD) [13,14]. Children with language disorders also exhibit pragmatic language problems at high rates (23–33%), further highlighting the associations between structural language impairments, pragmatic language impairments, and autism that have been argued in the literature for some time now [13,15].

Social functioning is considered a construct encompassing social skills, social cognition and peer functioning [16]. Peer functioning is thought to be comprised of two elements: peer status and friendships. Peer status relates to the degree that a child is accepted or rejected by their peers, and friendships are distinct from peer status, as they involve a relationship with a peer that is voluntary, mutual, and reciprocal. Friendships with peers are critical during childhood and adolescence, as they are associated with aspects of social and emotional development and adjustment, such as empathy, perspective taking and prosocial behaviours (e.g., [17]). Friendships during childhood and adolescence have been associated with fewer social problems, reduced internalising problems, and positive academic outcomes, and act as a protective factor against the negative outcomes associated with peer victimisation and bullying [18–20]. Conversely, failure to develop such peer relationships has long been associated with later emotional and behavioural problems [21].

**Fig 1:**
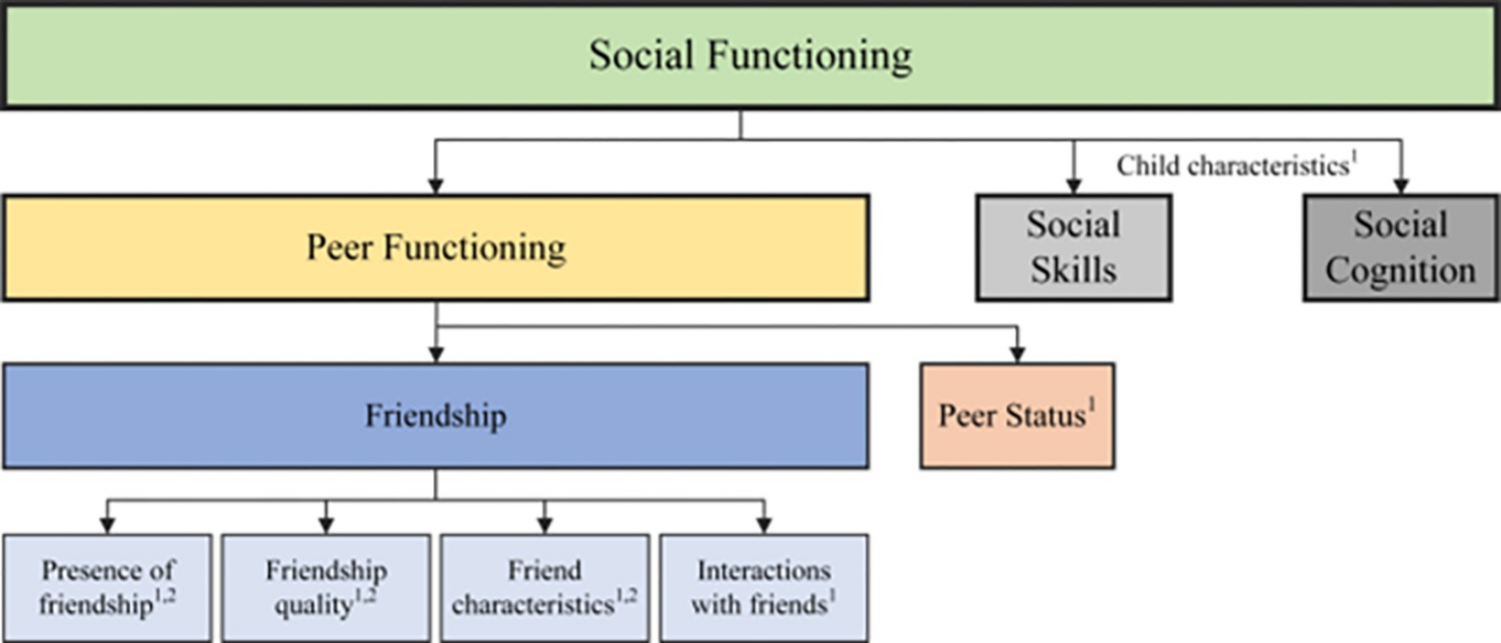
Conceptualisation of friendship in the context of social functioning according to Spender, Chen [22]. ^1^ Domain of friendship proposed by Bagwell and Schmidt [17], ^2^ Domain of friendship proposed by Hartup [23].

Two prominent models of friendship have been present in the literature for some time, and a recent review of friendships in children with ADHD synthesised these models to highlight elements of social functioning that are unique to friendship and attributes of friendship that are distinct from peer functioning and other aspects of social functioning [22]. Within the synthesis, Spender, Chen [22] note an alignment between the three domains of friendship (*having friends*, *friendship quality*, and *identity of friends*) proposed by Hartup [23] and the three elements of friendship (*presence of friendship*, *friendship quality*, and *characteristics of friends*) described by Bagwell and Schmidt [17]. The model developed by [17] also included the additional domains of *interactions with friends*, *child characteristics*, and *context of the friendship*, the latter two of which Spender, Chen [22] argue are more closely associated with the social skills and peer functioning aspects of social functioning, contributing to successful or unsuccessful friendships, but distinct from the construct of friendship itself. This study adopted the synthesised definition of friendship suggested by Spender, Chen [22], where friendship is a domain of peer functioning, associated with the presence and quality of friendship as well as the characteristics of and interactions with one’s friends (Fig 1).

The literature consistently identifies peer functioning, specifically friendship, as an area of social functioning that is a consistent challenge across children with ADHD, autism, ID, DLD and children with pragmatic language difficulties. Systematic reviews have documented significant social functioning differences between children with ADHD and typically-developing children, such as higher rates of victimisation or rejection by peers, lower rates of friendship, and reduced friendship quality and stability [1,22,24]. Similarly, children and young people with an intellectual disability are reported to have less contact with friends, have fewer friendships than their typically-developing peers, and spend more time alone [25]. A recent systematic review of the peer interactions of children with DLD found that children with DLD had higher levels of peer problems, lower levels of prosocial skills compared to children without DLD, and that children with DLD are less accepted than their peers [26]. Relationships with peers also manifest differently in autistic children compared to typically-developing peers, with evidence suggesting that children on the autism spectrum experience lower levels of friendship quality, and have the lowest rates of friendships among all disability groups [27].

Friendship challenges among children diagnosed with these neurodevelopmental disorders have been associated with the *child characteristics* and *peer status* aspects of social functioning. For example, children’s ability to communicate effectively, particularly pragmatic language, appears to be related to peer status and the building of friendships [28,29], which is pertinent for children with DLD who often struggle to access play and have poor conflict resolution skills [26], and children on the autism spectrum or with SPCD where challenges with social communication are hallmark features of these neurodevelopmental disorders [30]. Children on the autism spectrum have also been found to receive high rates of negative peer nominations and few positive peer nominations, which have been associated with externalising behaviours and social skills, respectively [31]. Social skills training is one area of adaptive functioning limitations associated with intellectual disability [30], and studies have found children with intellectual disabilities are often rejects or neglected by peers [32]. Children with ADHD often exhibit disruptive or inappropriate social behaviours and have difficulty with social cognition, perspective taking and social problem-solving [3,33], and a meta-analysis of associations between ADHD and social functioning found ADHD traits had a stronger association with peer functioning than with social skills or social cognition [16].

Many interventions have been developed to address *child characteristics* that are foundational to friendship challenges, and many systematic reviews have synthesised the evidence for different approaches regarding effectiveness in improving social skills and social cognition across these populations. In a systematic review of social communication interventions for children with DLD, the authors note several promising interventions for improving communication-related social interaction skills of children with DLD; however, further studies are required to establish effectiveness [34]. Fox, Dishman [35] synthesised the evidence for social skill interventions that involved peer interactions for children with ADHD, finding evidence that they improve play, pragmatic language and join attention skills and reduce inappropriate social behaviour. Similarly, systematic reviews of interventions for children with autism have found interventions that target social skills can have a positive effect on social communication abilities and play skills [36,37]. Social skills training interventions are the most common approach found in the literature for children with ID. They can have a positive effect on social skills ranging from eye contact to social problem-solving [38]. Being a relatively new diagnostic category in the DSM-5 with its own diagnostic challenges, systematic reviews on interventions for improving the social skills of children with SPCD are scant [39]. However, one recent intervention study reported positive changes in the conversation skills of children who appear to meet SPCD diagnostic criteria [40].

While the above summary of reviews is not exhaustive, it demonstrates that within the literature to date, interventions tend to be deemed effective if they have a measurable impact on the discrete range of social skills they set out to address. In the context of social functioning, such a narrow evaluation of intervention effect fails to consider that social functioning expands beyond *child characteristics*. This study is a systematic review and meta-analysis of social functioning interventions and their effect on children’s friendships with a common neurodevelopmental disorder (i.e., ADHD, autism, DLD, ID and SPCD). The specific aims of the review were to:

1. Identify intervention studies that measured friendship outcomes for children with ADHD, autism, DLD, ID or SPCD;
2. Describe the characteristics of interventions identified;
3. Appraise the methodological quality of intervention studies evaluating friendship outcomes for children with ADHD, autism, DLD, ID, and SPCD; and
4. Conduct a meta-analysis to evaluate the effectiveness of current interventions for improving friendships of children with ADHD, autism, DLD, ID or SPCD.

## Methods

The Preferred Reporting Items for Systematic Reviews and Meta-Analyses (PRISMA) statement and checklist guided the methodology and reporting of this study. The PRISMA statement guides the transparent reporting of systematic reviews and meta-analyses that evaluate the effect of interventions [41].

### Eligibility criteria

To be included in this systematic review and meta-analysis, studies were required to meet the following criteria: 1) participants were children and young people aged 0–18 years with a diagnosis of either autism, ADHD, intellectual disability, developmental language disorder, or social (pragmatic) communication disorder; 2) the study described an intervention that was expected to have an effect on the friendships of participants; 3) at least one aspect of friendship was measured as an intervention outcome, and 4) the study was a randomised controlled trial involving a comparison group of children who either received an alternative intervention, treatment as usual, or were waitlisted controls.

Given changes in the diagnostic definition of autism over time, children diagnosed with pervasive developmental disorder, Asperger’s syndrome, or Rett syndrome were included in the adopted definition of autism. Similarly, studies that included children described as having specific language impairment or developmental dysphasia/aphasia were also included, given the shifting terminology around developmental language disorders. Children with ADHD needed to meet the diagnostic criteria outlined in the DSM-III, DSM-IV, DSM -IV(R), or DSM-5. A diagnosis of global developmental delay/disability was considered within the adopted definition of intellectual disability.

There were no set criteria around the format of the interventions (i.e., who delivered the interventions, the setting, individual vs group-based interventions), the nature of friendship measurement (i.e., child-self report, peer-report, parent-report, educator-report), nor which aspect of friendship was measured (e.g., presence, quality, frequency). Studies were not included if they only investigated outcomes related to social skills associated with developing friendships (e.g., communication, problem-solving, and self-management skills). Original peer-reviewed journal articles of randomised control trials were included in this review. Cohort studies, case reports, reviews, conference abstracts, student dissertations and editorials were excluded. All studies were required to be published in English.

#### Information sources and search strategy

A literature search was performed in the following five electronic databases: CINAHL, Embase, Eric, PsycINFO, and PubMed. All publication dates up to the 28^th^ of May 2023 were included. To capture all literature on the subject of friendship and the selected diagnostic groups, subject headings related to the concepts of ADHD, autism, DLD, intellectual disability, SPCD, randomised controlled trials, and friendship were used in combination to identify the most recent publications (see Table 1 for search terms used per database). Limits were set for participant age to capture literature on children and young people only, and no limits were placed on the publication date. The content lists of past reviews involving friendship and the diagnostic groups of interest were screened to identify further publications. Additionally, reference lists of included studies were searched.

**Table 1:** Search strategies per database.

Database and Search Strategies	Number of records
**CINAHL:** ((MH "Autistic Disorder") OR (MH "Child Development Disorders, Pervasive") OR (MH "Pervasive Developmental Disorder-Not Otherwise Specified") OR (MH "Asperger Syndrome") OR (MH "Rett Syndrome") OR (MH "Attention Deficit Hyperactivity Disorder") OR (MH "Intellectual Disability") OR (MH "Developmental Disabilities") OR (MH "Language Disorders") OR (MH "Specific Language Impairment") OR (MH "Nonverbal Communication") OR (MH "Communicative Disorders") OR (Global AND developmental AND delay*) OR (Developmental AND Language AND Disorder*) OR (Social AND Communication) OR (Pragmatic AND Communication AND Disorder*) OR pragmatic* OR paralinguistic*) AND ((MH "Friendship") OR (MH "Peer Group") OR (MH "Interpersonal Relations") OR (MH "Social Environment") OR (MH "Social Adjustment") OR (MH "Social Participation") OR (MH "Social Networks") OR (MH "Social Networking") OR (MH "Social Inclusion") OR (MH "Social Cognition")) AND (MH “Randomized Controlled Trials”) *Limit Narrow by Subject Age*:*—all child*	114
**Embase:** (autism/ OR "pervasive developmental disorder not otherwise specified"/ OR Rett syndrome/ OR childhood disintegrative disorder/ OR attention deficit disorder/ OR intellectual impairment/ OR mental deficiency/ OR cognitive defect/ OR developmental delay/ OR developmental disorder/ OR language delay/ OR language development/ OR language disability/ OR language ability/ OR nonverbal communication/ OR communication disorder/ OR developmental language disorder/ OR paralanguage/ OR (Specific AND Language AND Impairment) OR (Social AND Communication) OR (Pragmatic AND Communication AND Disorder*) OR pragmatic* OR paralinguistic*) AND (friend/ OR friendship/ OR peer acceptance/ OR peer group/ OR social interaction/ OR social connectedness/ OR social participation/ OR social environment/ OR social adaptation/ OR social network/ OR social inclusion/ OR social cognition/ OR social acceptance/) AND (randomization/ OR randomized controlled trial/ OR controlled clinical trial/ OR “randomized controlled trial (topic)”/) *Limit to child <unspecified age>*	566
**Eric:** MAINSUBJECT.EXACT("Autism Spectrum Disorders") OR MAINSUBJECT.EXACT("Attention Deficit Disorders") OR MAINSUBJECT.EXACT("Attention Deficit Hyperactivity Disorder") OR MAINSUBJECT.EXACT("Moderate Intellectual Disability") OR MAINSUBJECT.EXACT("Mild Intellectual Disability") OR MAINSUBJECT.EXACT("Intellectual Disability") OR MAINSUBJECT.EXACT("Severe Intellectual Disability") OR MAINSUBJECT.EXACT("Developmental Disabilities") OR MAINSUBJECT.EXACT("Developmental Delays") OR MAINSUBJECT.EXACT("Language Impairments") OR MAINSUBJECT.EXACT("Oral Language") OR MAINSUBJECT.EXACT("Receptive Language") OR MAINSUBJECT.EXACT("Language Proficiency") OR MAINSUBJECT.EXACT("Naming") OR MAINSUBJECT.EXACT("Communication Problems") OR MAINSUBJECT.EXACT("Verbal Communication") OR MAINSUBJECT.EXACT("Expressive Language") OR MAINSUBJECT.EXACT("Communication Disorders") OR MAINSUBJECT.EXACT("Nonverbal Communication") OR MAINSUBJECT.EXACT("Language Ability (1966 1980)") OR MAINSUBJECT.EXACT("Language Acquisition") OR MAINSUBJECT.EXACT("Pragmatics") OR MAINSUBJECT.EXACT("Paralinguistics") OR (Developmental AND Language AND Disorder*) OR (Specific AND Language AND Impairment*) OR (Social AND Communication AND Disorder*) OR (Pragmatic AND Communication AND Disorder*)) AND (MAINSUBJECT.EXACT("Friendship") OR MAINSUBJECT.EXACT("Peer Relationship") OR MAINSUBJECT.EXACT("Peer Groups") OR MAINSUBJECT.EXACT("Peer Influence") OR MAINSUBJECT.EXACT("Peer Acceptance") OR MAINSUBJECT.EXACT("Interpersonal Relationship") OR MAINSUBJECT.EXACT("Social Cognition") OR MAINSUBJECT.EXACT("Social Relations (1966 1980)") OR MAINSUBJECT.EXACT("Social Environment") OR MAINSUBJECT.EXACT("Social Adjustment") OR MAINSUBJECT.EXACT("Social Networks") OR MAINSUBJECT.EXACT("Social Cognition")) AND (RCT OR (Randomized AND Controlled AND Trial) OR (Randomised AND Controlled AND Trial) OR (Randomized AND Clinical AND Trial) OR (Randomised AND Clinical AND Trial) OR (Controlled AND Clinical AND Trial))	32
**PsycINFO:** (autism/ OR aspergers syndrome/ OR pervasive developmental disorders/ OR rett syndrome/ OR attention deficit disorder with hyperactivity/ OR intellectual development disorder/ OR "intellectual development disorder (attitudes toward)"/ OR cognitive impairment/ OR developmental disabilities/ OR language delay/ OR language development/ OR language disorders/ OR oral communication/ OR language proficiency/ OR verbal communication/ OR verbal comprehension/ OR nonverbal communication/ OR communication disorders/ OR specific language impairment/ OR pragmatics/ OR (Developmental AND Language AND Disorder*) OR (Social AND Communication AND Disorder*) OR (Pragmatic AND Communication AND Disorder*)) AND (friendship/ OR peer relations/ OR classmates/ OR social functioning/ OR social inclusion/ OR social acceptance/ OR social adjustment/ OR social cognition/ OR social connectedness/ OR social environments/ OR social networks/ OR social interaction/) AND (RCT OR (Randomized AND Controlled AND Trial) OR (Randomised AND Controlled AND Trial) OR (Randomized AND Clinical AND Trial) OR (Randomised AND Clinical AND Trial) OR (Controlled AND Clinical AND Trial)) *Limit to (childhood <birth to age 12 yrs> or adolescence <age 13 to 17 yrs>)*	52
**PubMed:** ("Autistic Disorder"[Mesh] OR "Child Development Disorders, Pervasive"[Mesh] OR "Rett Syndrome"[Mesh] OR "Asperger Syndrome"[Mesh] OR "Attention Deficit Disorder with Hyperactivity"[Mesh] OR "Intellectual Disability/education"[Mesh] OR "Intellectual Disability/nursing"[Mesh] OR "Intellectual Disability/prevention and control"[Mesh] OR "Intellectual Disability/psychology"[Mesh] OR "Intellectual disability/rehabilitation"[Mesh] OR "Intellectual Disability/therapy"[Mesh] OR "Cognitive Dysfunction/diet therapy"[Mesh] OR "Cognitive Dysfunction/nursing"[Mesh] OR "Cognitive Dysfunction/prevention and control"[Mesh] OR "Cognitive Dysfunction/psychology"[Mesh] OR "Cognitive Dysfunction/rehabilitation"[Mesh] OR "Cognitive Dysfunction/therapy"[Mesh] OR "Language Development Disorders"[MeSH Terms] OR "Language Disorders"[MeSH Terms] OR "Language Development"[MeSH Terms] OR "Specific Language Disorder"[MeSH Terms] OR "Communication Disorders"[MeSH Terms] OR "Nonverbal Communication"[Mesh] OR "Specific Language Impairment 4" [Supplementary Concept] OR "Social Communication Disorder"[Mesh] OR (Developmental AND Language AND Disorder*) OR (Specific AND Language AND Impairment*) OR (Pragmatic AND Communication AND Disorder*) OR pragmatic* OR paralinguistic* OR (social AND communication)) AND ("Friends"[Mesh] OR "Peer Influence"[Mesh] OR "Peer Group"[Mesh] OR "Interpersonal Relations"[Mesh] OR "Social Interaction"[Mesh] OR "Social Adjustment"[Mesh] OR "Social Cognition"[Mesh] OR "Social Networking"[Mesh] OR "Social Participation"[Mesh] OR "Social Inclusion"[Mesh]) AND (“Randomized Controlled Trial” [Publication Type] OR “Randomized Controlled Trials as Topic”[Mesh] OR “Controlled Clinical Trial” [Publication Type] OR “Pragmatic Clinical Trials as Topic”[Mesh]) *Limit Child*: *birth-18 years*	837

#### Selection process

Records retrieved from the electronic databases were first screened for duplicates. After duplicates were removed, all abstracts were screened against the selection criteria. The full text of the studies was then retrieved and assessed to determine the final studies to be included in the review. Two reviewers performed all screening stages independently to ensure record and study selection accuracy. Where reviewer disagreement occurred, they met and discussed the article with a third reviewer to achieve consensus on the eligibility of articles.

#### Data collection process and data items

Data from the selected articles were collected into data extraction tables developed to address the aims of the systematic review and the meta-analysis. The use of data extraction tables ensured that the same data characteristics were extracted from all included papers [42]. One reviewer extracted data into each table, after which a second author checked the retrieved data for accuracy. Data extraction tables were developed to facilitate an understanding of each study’s design, target populations, and intervention characteristics. Data items within the extraction tables therefore collated data about each study’s design (groups, sample sizes, participant ages, inclusion criteria, friendship outcome measures, results reported) and each intervention’s characteristics (areas of social participation, intervention techniques, setting, facilitator of the intervention, the nature of peer and parent involvement, the duration and frequency of intervention sessions).

#### Risk of bias assessment

The revised Cochrane Risk of Bias Tool for Randomised Trials (RoB2 tool) was used to assess the methodological quality of the included studies [43]. The RoB2 tool assesses five domains where potential bias can occur. The domains include the randomisation process (3 items), deviations from intended interventions (7 items), missing outcome data (4 items), measurement of the outcome (5 items), and selection of the reported result (3 items). After scoring the items within each domain, an assessment is made of the overall risk of bias present within the domain, resulting in a rating per domain of either “Low”, “High”, or “Some concerns”. An overall risk of bias rating is then given to the study based on the risk of bias present within each domain. For this study, two reviewers independently completed the RoB2 checklist for all included studies and resolved disagreements by discussion until consensus was reached. Reviewers have no affiliation with any authors of the included studies; therefore, the level of bias is reduced regarding the extraction of data and ratings of study quality [36].

#### Meta-analysis

To conduct the meta-analysis, statistics related to outcome measurement were extracted into a meta-analysis table (e.g., pre-and post-mean values, standard deviations, sample size). Studies judged as having a “High” overall risk of bias were excluded from the meta-analysis to reduce the risk of bias within the results of this study. Data were extracted for meta-analysis for both the experimental and control groups of all studies to facilitate between-group analyses. Data items related to intervention characteristics were created to facilitate subgroup analysis according to the following categorisation: participant diagnosis (ADHD, autism, DLD, ID, SPCD), level of peer-inclusion in the intervention (peer-mediation, peer proximity, peer involvement) [44], outcome measure respondent, and facilitator. Efforts were made to contact authors when desired data were not reported and were needed for meta-analysis calculations.

Extracted data from the included studies were entered into Comprehensive Meta-Analysis, Version 4.0, for meta-analysis. First, within-groups analysis evaluated the overall intervention effect on friendship from all interventions across all studies. Next, the between-group intervention effect on friendship was assessed by comparing intervention-group outcomes with control-group outcomes. Finally, studies were grouped based on participant diagnosis, type of peer inclusion in the intervention, facilitator, and outcome measure respondent to facilitate sub-group analysis. Within- and between-group analyses were conducted for each study sub-grouping to understand whether intervention effects differed based on grouping variables.

Random-effects modelling was used to generate effect sizes, given the likelihood that included studies did not have the same true effect due to the variability in intervention characteristics, participant characteristics, sampling, skills targeted, and outcome measure utilised; therefore, considering real differences in the treatment effect of each study and the potential variance of estimates of treatment effect [45]. The Hedges-*g* formula for standardised mean difference (SMD) with a confidence interval of 95% (95% CI) was used to measure effect size [46]. Effect sizes were interpreted using Cohen’s-*d* conventions: 0.2–0.49 was considered a small effect, 0.5–0.79 was considered a moderate effect, and ≥0.8 reflects a large effect [47]. Prediction intervals were calculated to measure heterogeneity within the meta-analysis [48].

The Classic Fail-Safe N was used to assess publication bias by calculating the number of additional studies that, if included in the analysis, would nullify the measured effect (N). If the value of N is large, relative to the number of observed studies, the meta-analyses are unlikely to be compromised by publication bias as the likelihood of there being many unpublished low-effect or negative-effect studies is low [49].

## Results

### Study selection

**Fig 2:**
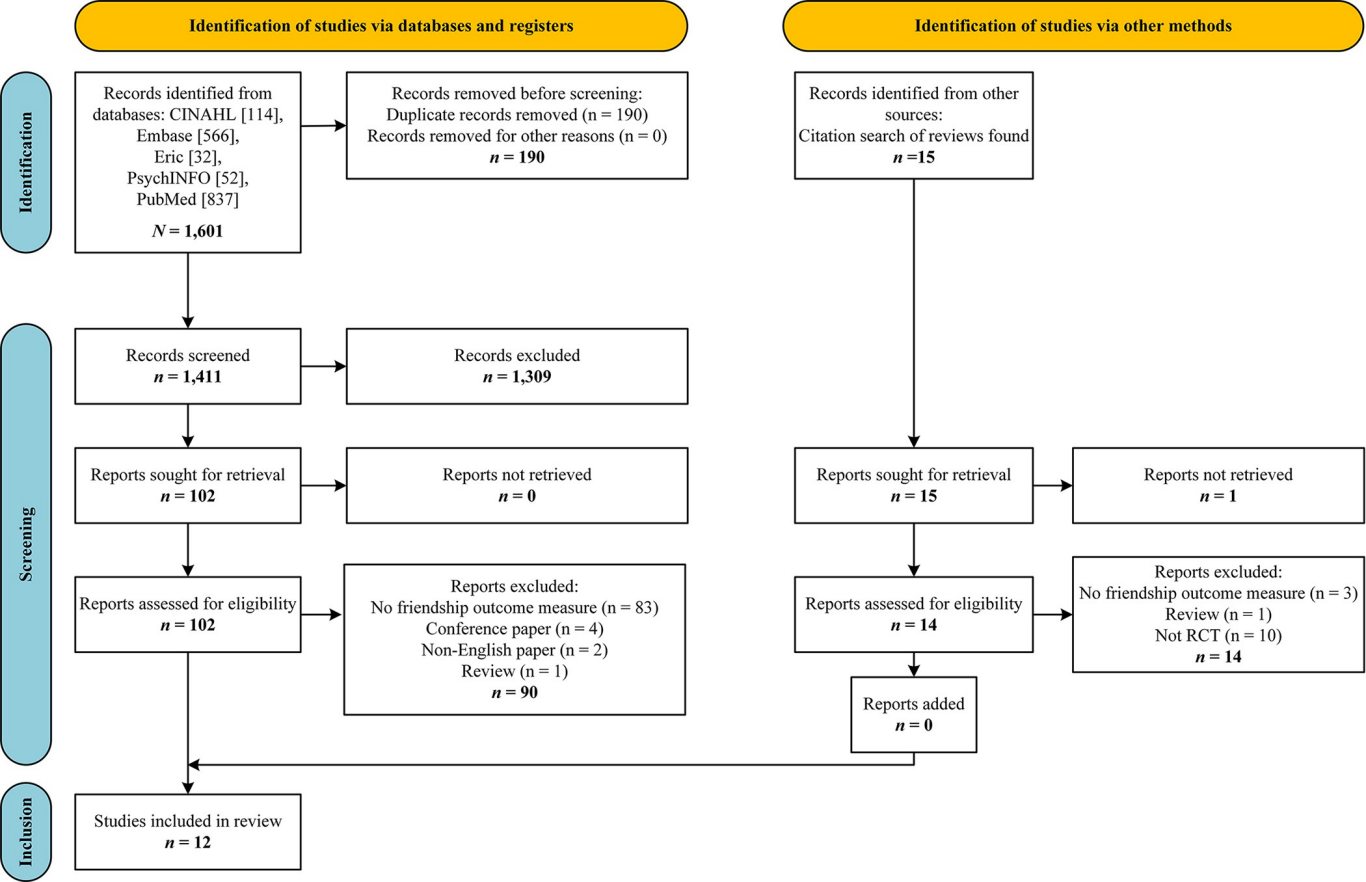
PRISMA flow diagram of record screening process.

Overall, 1,601 records were retrieved from the four electronic databases searched. After duplicates were removed, 1,411 abstracts were screened by two independent reviewers and 1,309 records were excluded from further screening. A total of 102 full-text articles were accessed and assessed for eligibility, with reviewers identifying 12 studies meeting eligibility criteria for inclusion in the review. No additional study meeting the inclusion criteria was retrieved from hand-searching previous friendship reviews and reference lists of included articles. A total of 12 studies reporting on 15 different interventions were included in the review. Fig 2 details the search and screening process.

### Description of studies

#### Participants

Table 2 provides a detailed description of the studies included in this review. A total of 683 children with a neurodevelopmental disorder and 190 typically-developing children participated in the 12 studies included in the systematic review. Participants in 10 studies had a diagnosis of either autism (*n* = 6 studies) or ADHD (*n* = 4 studies), and two studies included children with autism, ID, or co-occurring autism and ID. Children’s diagnoses were confirmed either using a standardised assessment or by reviewing diagnostic reports. One study involving children with ADHD provided rates of co-occurring symptoms of anxiety, depression, and oppositional defiant disorder for participants within their studies [50–52]. Three studies involving children with ADHD also included data on typically-developing participants (*n* = 190), and no studies involving children with DLD, ID or SPCD were found.

**Table 2:** Summary of included studies.

Reference Methodological quality (RoB2)	Intervention Comparison condition(s)	Sample size (Mean Age[yrs] ± SD/ Grade Range)	Inclusion Criteria	Friendship Outcome Measure; Score (Respondent)	Results
Asmus, Carter [53] Low risk	Peer Network Intervention	*n* = 47 (9^th^-12^th^ Grade)	Significant cognitive impairments, or received special education for ID or autism Enrolled in ≥1 general education class with education support	Social Connections and Relationships Assessment; Friendship gains (Educator)	Post-intervention: Significant main effect of group (*d* = 1.39). Follow-up: Effect maintained at one semester follow-up (*d* = 0.38); No significant effect at two semester follow-up.
	Treatment as usual	*n* = 48 (9^th^-12^th^ Grade)			
				Social Connections and Relationships Assessment; Friendship gains (Parent)	Post-intervention: No significant main effect of group (*d* = 0.20); Follow-up: No significant effect at one or two semester follow-up.
Brock, Dueker [54] High risk	Practitioner-facilitated Peer-Implemented Pivotal Response Training	*n =* 6 (8-12yrs)	Diagnosis of autism Not frequently interacting with peers at recess	Social validity survey question: *Do you consider the trained peers to be your friends*? (Child)	Post-intervention: 83% considered trained peers to be their friends. Follow up: NA
	Treatment as usual	*n =* 5 (8-12yrs)			
				5-point Likert scale: *As a result of this strategy*, *the student with autism made more friends*? (Educator)	Post-intervention: Mean = 4.2 (SD = 1.3) Follow up: NA
Carter, Asmus [55] Low risk	Peer support group	*n* = 51 (9^th^-12^th^ Grade)	Received special education services under the categories ID or autism, or qualified for alternate assessment, enrolled in ≥1 general education class, have individually assigned education support	Social Connections and Relationships Assessment; Friendship gains (Educator)	Post-intervention: Significant main effect of group on friendship gains at school (*d* = 1.02) Follow up: NA
	Treatment as usual	*n =* 48 (9^th^-12^th^ Grade)			
Kasari, Rotheram-Fuller [56] Low Risk	Child-assisted (CHILD) intervention	*n =* 15 (8.23 **±**1.48)	Met autism criteria on the ADI-R and ADOS; in a regular education classroom for at least 80% of the school day; aged 6–11yrs; in grades 1–5; IQ score ≥65 on the WISC-IV; no additional diagnoses	The Friendship Survey; Reciprocal friendship nominations (Child)	Post-intervention: No significant between groups difference Follow up: No significant between-group difference
	Peer-mediated (PEER) intervention	*n =* 15 (7.60 **±**1.35)			
	PEER and CHILD	*n* = 15 (8.67 ±1.68)			
	No intervention	*n* = 15 (8.07 ±1.69)			
Laugeson, Frankel [57] Low Risk	PEERS®	*n =* 17 (14.6 ±1.3)	Aged 13-17yrs; social problems reported by parent; diagnosis of “high functioning autism”, Asperger’s Disorder, or PDDNOS; fluent in English; parent/family member fluent in English; K-BIT-2 verbal IQ ≥ 70; no history of major mental illness, hearing, visual, or physical impairments	Friendship Qualities Scale; Total score (Child)	Post-intervention: Significant effect of group favouring PEERS® (*p* <0.05) Follow up: NA
	Waitlisted control	*n =* 16 (14.6 ±1.6)			
Lerner and Mikami [58] Low Risk	Sociodramatic Affective Relational Intervention (SDARI)	*n =* 7 (10.86 ±1.68)	“High functioning autism” diagnosis made by a licensed professional	Sociometric nominations; Reciprocal friendship nominations (Child)	Post-intervention: No significant difference between groups over time. Significant effect of time for both groups with large effect (*ŋ*^2^ = 0.31) Follow up: NA
	Skillstreaming	*n =* 6 (11.13 ±1.63)			
Locke, Shih [59] Low Risk	Remaking Recess (RR)	*n =* 14 (9.0; 1^st^–5^th^ Grade)	Autism diagnosis; referred by school administrators; IQ score ≥ 65 in school records; in a general education classroom (K-5^th^ grade) for ≥80% of the school day.	Friendship Survey; Received friendship nominations (Child).	Post-intervention: No significant increase across both conditions. No between-group differences over time. Follow up: NA
	Remaking Recess with implementation support (RR+)	*n* = 17 (8.6; K-5^th^ Grade)			
Mikami, Lerner [50] Low Risk	Parental Friendship Coaching	*n =* 32 (8.28 ±1.30)	Children with ADHD: exceeded clinical cut-offs for ADHD on parent and educator CSI; diagnosis verified with K-DAS	5-point Likert scale: Extent to which child’s friendships had changed since the study period (Parent)	Post-intervention: no measurement of friendship Follow up: Significant improvement in friendships for PFC group compared to no intervention ADHD group: *F* (1, 110) = 27.62, *p*<0.01
	No treatment control (ADHD)	*n =* 30 (8.23 ±1.14)			
	No treatment, age-matched TD	*n =* 62 (8.23 ±1.19)	Did not meet criteria for ADHD on parent or educator CSI and the K-SADS.		
Mikami, Griggs [51] Low Risk	MOSAIC then COMET	*n =* 24; 12 per group (8.15 ±0.79)	≥6 symptoms of inattention or hyperactivity/impulsivity on parent and educator CSI; ≥3 items of peer impairment endorsed by parents or educators; < 50% of peers rated as liking them.	Sociometric nominations; Reciprocal friendship nominations (Child)	Post-intervention: Significantly more friendship nominations for children with ADHD following MOSAIC than COMET (ŋ_p_^2^ = 0.34) Follow-up: NA
	COMET then MOSAIC				
	TD Participants (MOASIC and COMET)	*n* = 113 (8.15 ±0.79)	≤3 symptoms of inattention or hyperactivity/impulsivity on parent and educator CSI; ≤1 item of peer impairment endorsed by parents or educators; >50% of peers rated as liking them		
Mikami, Normand [52] Low Risk	Parental Friendship Coaching	*n =* 84 (8.74 ±1.60)	≥6 symptoms of inattention or hyperactivity/impulsivity by parent K-SADS or educator CSI	Friendship Quality Questionnaire-Short; Positive friendship quality, Negative friendship quality (Parent, Child, Friend, Parent of Friend composite)	Post-intervention: No significant effect of group. Follow up: No significant effect of group
	CARE	*n =* 88 (8.35 ±1.49)			
				Friendship quality on observation; Positive friendship quality, Negative friendship quality (Independent observer)	Post-intervention: No significant effect of group. Follow up: No significant effect of group
Schohl, Van Hecke [60] Low Risk	PEERS®	*n =* 29 (14 ±1.28)	Aged 11-16yrs; social problems reported by parent; fluent English; parent/family member fluent in; no history of major mental illness, hearing, visual, or physical impairments; diagnosis of either “high functioning autism”, Asperger’s Disorder, or PDDNOS confirmed by ADOS; K-BIT-2 verbal IQ ≥ 70.	Friendship Qualities Scale; Total score (Child)	Post-intervention: No significant between-group difference. Follow up: NA
	Waitlist control	*n =* 29 (13.31 ±1.65)			
Whalen, Henker [61] Low Risk	0.6mg/kg methylphenidate	*n* = 25 (9.1; range 6.3–12.4yrs)	Diagnosis of hyperactivity, ADHD, or ADHD from referring physicians; no signs of intellectual disability or gross neurological dysfunction.	Sociometric nominations; Received friendship nominations (Child).	Post-intervention: Significant treatment effect for “best friend” nominations; positive effect increasing between placebo, 0.3mg/kg, and 0.6mg/kg conditions Follow-up: NA
	0.3mg/kg methylphenidate				
	Placebo				
	TD participants	*n* = 15 (8.7; range 7.0–10.3yrs)	No known intellectual, behavioural or academic problems		

*Notes*. No studies in this review included children with developmental language disorder (DLD), social (pragmatic) communication disorder (SPCD) or intellectual disability (ID); PEERS® = Program for the Education and Enrichment of Relational Skills; CARE = Coping with ADHD through Relationships and Education; MOSAIC = Making of Socially Accepting Inclusive Classrooms; COMET = Contingency Management Training; CHILD = Child Assisted Approach; PEER = Peer Mediated Approach; RR = Remaking Recess; RR+ = Remaking Recess with implementation support; PFC = Parental Friendship Coaching; SDARI = Sociodramatic Affective Relational Intervention; ADHD = attention-deficit hyperactivity disorder; TD = typically-developing; yrs = years; SD–standard deviation; *d* = Cohen’s-*d*; NA = not applicable.

Participants’ ages ranged from 6–17 years of age. Eight studies included children with a neurodevelopmental disorder in middle childhood (i.e., 6–12 years of age; *n* = 398), with the remaining four studies targeting adolescents (*n* = 285). All children with ADHD who received an intervention were younger than 13 years old (4 studies), whereas studies that included autistic children evaluated interventions for children across middle-childhood (4 studies) and adolescence (4 studies). Participant characteristics and inclusion criteria per study are reported in Table 2.

#### Study groups and research designs

Sample sizes within the 12 studies ranged from 11 to 172. Most commonly, studies (*n* = 5; 41.7%) recruited fewer than 50 participants, and four studies (33.3%) included between 50 and 100 participants. Two studies (16.7%) had between 100 and 150 participants, and one study (8.3%) had more than 150 participants.

All studies randomised participants to groups, and most studies (*n* = 8; 66.7%) involved two groups: an intervention group who received an intervention of interest, and a control group who were either waitlisted (*n* = 2), received treatment as usual (*n* = 3), or received another intervention focused on social interactions (*n* = 3). Mikami, Lerner [50] randomised participants with ADHD into two groups (intervention and non-intervention controls) and included an additional age-matched comparison group of typically-developing children. Kasari, Rotheram-Fuller [56] randomised participants into four groups: three different treatment groups and one control group that received no intervention. The two remaining studies used a cross-over design. Whalen, Henker [61] randomised participants into three groups, and participants were involved in three treatment groups across the study, while Mikami, Griggs [51] randomised participants into two groups to participate in two interventions in contrasting order.

#### Outcome measurement

Most studies (*n* = 8; 66.7%) reported measures of friendship at at-least two time points (pre- and post-intervention), with two of the eight also reporting follow-up measurements ranging from three to six months. Mikami, Griggs [51], Brock, Dueker [54], and Whalen, Henker [61] measured friendship at post-intervention only, and Mikami, Lerner [50] measured friendship at one-month follow-up only.

#### Measures of friendship

Sociometric nominations were the most common method of friendship measurement, occurring in seven studies. Reciprocal friend nominations via the children themselves were measured in 4 studies, received friendship nominations from children were measured in one study, and parents and educators nominated friends in two studies. Three studies utilised a standardised measure of friendship quality, completed by either the child (2 studies) or a combination of self-, peer-, parent-, and parent-of-the-peer-reports (1 study). One further study also utilised an observational measure of friendship quality during play interactions with a friend.

### Interventions

Fifteen interventions were included across the 12 studies. Of the ten interventions for autistic children, most were delivered at school (*n* = 7) or in an after-school group setting (*n* = 2). One intervention, PEERS®, was delivered in a clinic setting through a group format. School-based interventions tended to involve didactic instruction to the regular peers of target children, who then mediated the delivery of the intervention by modelling targeted social skills and behaviours, with some also including didactic instruction in social skills to autistic children also. The after-school groups (SDARI, Skillstreaming) and the clinic-based intervention (PEERS®) involved instruction to the target children and peer involvement in activities to facilitate practice of targeted social skills. Parent involvement was included only in the clinic-based intervention. School-based interventions occurred either once or twice a week or daily. Clinic-based interventions were delivered weekly. Peer functioning and social skills were the targets for all interventions for autistic children, with social cognition skill development also included in five interventions.

Of the five interventions for children with ADHD, two were delivered in a clinic setting, and three were part of summer camp programs. Parents of children with ADHD were the participants in both clinic-based interventions (Parental Friendship Coaching [PFC], CARE), where facilitators provided psycho-education or coaching and parents implemented strategies with their child at home. The three summer camp programs involved three different intervention approaches. Mikami, Griggs [51] trialled contingency behaviour management techniques within one intervention approach (COMET), and contingency behaviour management techniques combined with facilitator modelling of social validation and positive attention in another approach (MOSAIC), while Whalen, Henker [61] trialled stimulant medication at different dosages. Clinic sessions for parents occurred weekly for 90 minutes, and summer camp approaches were delivered daily. Again, peer functioning was the target of all interventions for children with ADHD, and social skills were included in PFC, CARE, COMET, and MOSAIC. Stimulant medication was administered to have a global effect on behaviour that included but was not limited to behaviours related to social functioning. A summary of all interventions is provided in Table 3.

**Table 3:** Characteristics of friendship interventions.

Intervention Reference	Social functioning component	Intervention techniques	Setting	Interventionist	Peer Inclusion	Parent Involvement	Session Frequency/Duration
**Interventions involving children on the autism spectrum**							
**Peer Network Intervention** Asmus, Carter [53]	Social Skills Peer Functioning	Facilitator-led didactic instruction to peers Peer modelling to target children	School	Educators & Clinician	Peer-mediation	None	Approximately once per week for an average length of 56.10 minutes (*SD* = 18.7).
**Practitioner-facilitated Peer-Implemented Pivotal Response Training** Brock, Dueker [54]	Social Skills Social Cognition	Facilitator-led didactic instruction to peers Peer modelling to target children	School	Educators	Peer-mediation	None	A day-to-day basis for a minimum of 5 weeks.
**Peer support group** Carter, Asmus [55]	Social Skills Peer Functioning Social Cognition	Facilitator-led didactic instruction Facilitator modelling and feedback to peers Peer modelling to target children	School	Educators	Peer-mediation	None	A day-by-day basis (*M* = 8.4 weeks, SD = 2.4).
**Child-assisted (CHILD) intervention** Kasari, Rotheram-Fuller [56]	Social Skills Peer Functioning	Facilitator-led didactic instruction to target children	School	Clinician	Peer involvement	None	12 sessions over 6 weeks (20 minutes per session).
**Peer-mediated (PEER) intervention** Kasari, Rotheram-Fuller [56]	Social Skills Peer Functioning Social Cognition	Facilitator-led didactic instruction to peers Peer-led didactic instruction, role-playing, and modelling to target children	School	Clinician	Peer-mediation	None	12 sessions over 6 weeks (20 minutes per session).
**Sociodramatic Affective Relational Intervention (SDARI)** Lerner and Mikami [58]	Social Skills Peer Functioning Social Cognition	Facilitated activities to elicit practice of targeted social skills	After-school groups	Clinician	Peer involvement	None	Once per week for 4 weeks (90-minute meetings, including two 40-minute sessions).
**Skillstreaming** Lerner and Mikami [58]	Social Skills Peer Functioning Social Cognition	Facilitator-led Didactic instruction	After-school groups	Clinician	Peer involvement	None	Once per week for 4 weeks (90-minute meetings, including two 40-minute sessions).
**Remaking Recess (RR)** Locke, Shih [59]	Social Skills Peer Functioning	Didactic instruction in intervention techniques to educators Facilitator modelling, feedback to educators Scaffolding target child’s social engagement with peers Facilitator-led didactic instruction to target child Facilitator-led didactic instruction to peers	School	Clinician & Educator	Peer mediation	None	Twice per week for 6 weeks (30–45-minute sessions)
**Remaking Recess with implementation support (RR+)** Locke, Shih [59]	Social Skills Peer Functioning	See RR above Facilitator support to school to address barriers to implementation	School	Clinician & Educator	Peer mediation	None	Twice per week for 6 weeks (30–45-minute sessions). 3 additional implementation support sessions over 6 weeks
**PEERS®** Laugeson, Frankel [57], Schohl, Van Hecke [60]	Social Skills Peer Functioning	Facilitator-led didactic instruction Facilitator modelling, role play, feedback Psycho-education for parents/caregivers	Clinic	Clinician	Peer involvement	Psycho-education sessions Implementation of strategies at home	12–14 sessions once per week (90-minute sessions).
**Interventions involving children with ADHD**							
**Parental Friendship Coaching (PFC)** Mikami, Lerner [50], Mikami, Normand [52]	Social Skills Peer Functioning	Facilitator-led didactic instruction and role play to parents/caregivers	Clinic	Clinician (Parent-secondary interventionist)	None	Coaching child in targeted friendship behaviours	8–10 sessions once per week (90-minutes per session).
**CARE** Mikami, Normand [52]	Social Skills Peer Functioning	Psycho-education for parents/caregivers	Clinic	Clinician (Parent-secondary interventionist)	None	Attendance at CARE sessions	10 sessions once per week (90 minutes per session).
**MOSAIC** Mikami, Griggs [51]	Social Skills Peer Functioning	Contingent behaviour management Facilitator modelling of social validation, and positive attention	Summer camp	Educator	Peer involvement	None	5 days per week for 2 weeks (6 hours per day).
**COMET** Mikami, Griggs [51]	Social Skills	Contingent behaviour management	Summer camp	Educator	Peer involvement	None	5 days per week for 2 weeks (6 hours per day).
**Methylphenidate** Whalen, Henker [61]	Peer Functioning	Stimulant medication	Summer camp	Clinician	Peer involvement	None	Twice daily for 5 weeks.

*Note*. PEERS® = Program for the Education and Enrichment of Relational Skills; CARE = Coping with ADHD through Relationships and Education; MOSAIC = Making of Socially Accepting Inclusive Classrooms; COMET = Contingency Management Training; CHILD = Child Assisted Approach; PEER = Peer Mediated Approach; RR = Remaking Recess; RR+ = Remaking Recess with implementation support; PFC = Parental Friendship Coaching; SDARI = Sociodramatic Affective Relational Intervention; M = mean; SD = standard deviation.

### Risk of bias assessment

The methodology of all included studies included were assessed for bias using the RoB2 critical appraisal tool [43]. Of the 12 studies assessed, 11 were rated as having a low risk of bias overall and across all five domains of risk. The study by Brock, Dueker [54] was identified as having a high risk of bias overall, due to outcome measurement methods. All other domains of the Brock, Dueker [54] study were assessed as having a low risk of bias. Overall ratings per study are presented in Table 2, and domain-level ratings for each study are presented in S1 Table.

### Meta-analysis

Six of the 12 studies assessed for eligibility in the meta-analysis were excluded. Four studies did not report the necessary data to conduct the meta-analysis [50,53,55,56], and one study was judged as having a high risk of bias [54]. The decision was made to also exclude Whalen, Henker [61] to reduce heterogeneity within the meta-analysis; Whalen, Henker [61] evaluated a pharmacological intervention, where the remaining six studies were more closely aligned in their approaches (i.e., psychosocial, behavioural). Where studies reported more than one outcome measure of friendship, pooled means and standard deviations were derived for the menta-analysis to ensure only one effect size was calculated per study. Nine interventions were included in the within-group analysis of the six studies in the meta-analysis.

#### Overall, within-group analysis

**Fig 3:**
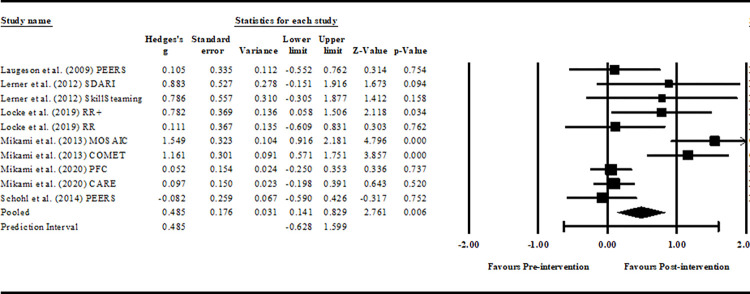
Pre-post within-group comparison. *Notes*. PEERS® = Program for the Education and Enrichment of Relational Skills; SDARI = Sociodramatic Affective Relational Intervention; RR+ = Remaking Recess with implementation support; RR = Remaking Recess; MOSAIC = Making of Socially Accepting Inclusive Classrooms; COMET = Contingency Management Training; PFC = Parental Friendship Coaching; CARE = Coping with ADHD through Relationships and Education; Hedges-g: 0.2–0.49 = small effect, 0.5–0.79 = moderate effect, and ≥0.8 = large effect.

The pooled intervention effects for friendship across all interventions were small to moderate (*z* = 2.761, *p* = 0.006, *g =* 0.485, 95% CI = 0.141–0.829), with a prediction interval of -0.628 to 1.599. Pre-post-intervention effects varied greatly across the studies, ranging from -0.082 to 1.549. Five interventions (54.5%) produced a negligible effect (*g* = - 0.082–0.111). Skillstreaming and Remaking Recess with Intervention Support (RR+) produced effects that approached large effect sizes (0.5 < Hedges’ *g* = 0.782–786), and SDARI, MOSAIC and COMET produced conclusively large effects (Hedges’ *g* > 0.8). See Fig 3 for full results. The classic fail-safe N for the within-groups analysis was 50, suggesting a low risk of publication bias for the analysis.

#### Overall, between-group analysis

**Fig 4:**
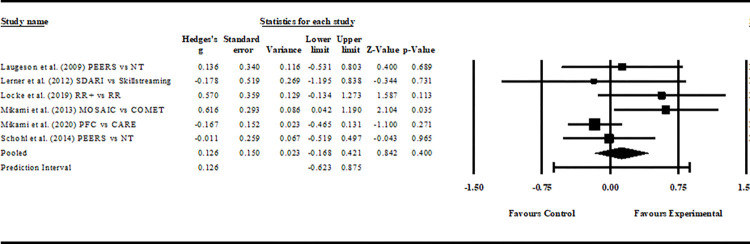
Between-group comparison. *Notes*. PEERS® = Program for the Education and Enrichment of Relational Skills; NT = no treatment; SDARI = Sociodramatic Affective Relational Intervention; RR+ = Remaking Recess with implementation support; RR = Remaking Recess; MOSAIC = Making of Socially Accepting Inclusive Classrooms; COMET = Contingency Management Training; PFC = Parental Friendship Coaching; CARE = Coping with ADHD through Relationships and Education; Hedges-g: 0.2–0.49 = small effect, 0.5–0.79 = moderate effect, and ≥0.8 = large effect.

The pooled intervention effect between intervention and comparison groups was not significant (*z =* 1.206, *p =* 0.400, *g =* 0.215, 95% CI = -0.168–0.421), with a prediction interval of -0.623 to 0.875. MOASIC was the only intervention to produce a significant effect compared to the control comparison (*p* = 0.035, *g* = 0.616, 95% CI = 0.042–1.190). RR+ produced a moderate effect compared to Remaking Recess (RR) without the intervention support, but the difference was not significant (*p* = 0.113, *g* = 0.570, 95% CI = -0.134–1.273). All other interventions produced either negligible (Hedges’ *g* ≤ 0.2) or negative effects (i.e., outcome favours the control comparison; Hedges’ *g* ≤ 0). Fig 4 contains all between-group results. The fail-safe N was irrelevant, given that the result was not significant.

#### Within subgroup analyses

Findings from the within-group meta-analysis (Table 4) indicated that only interventions delivered to participants with ADHD had a significant, positive effect (*p* = 0.040, *g* = 0.655, 95% CI = 0.031–1.280). Interventions that utilised peer involvement had larger effect sizes than interventions with peer mediation or without any peer engagement (*p* = 0.018, *g* = 0.716, 95% CI = 0.123–1.310). Additionally, interventions facilitated by educators had a significant, large effect (*p* < 0.001, *g* = 1.341), with parent- or clinician-facilitated interventions producing negligible effects. Comparisons between outcome measures indicated significant effects for child self-report measures only (*p* = 0.006, *g* = 0.671, 95% CI = 1.149–2.745).

**Table 4:** Within and between-groups meta-analyses comparing effects for subgroups of included studies.

		Within-groups						Between-Groups				
Subgrouping	*n*	*Hedges’ g*	Lower limit	Upper limit	Z-value	*p-value*	*n*	*Hedges’ g*	Lower limit	Upper limit	Z-value	*p-value*
**Diagnosis**												
ADHD	4	0.655	0.031	1.280	2.056	0.040*	2	0.184	-0.579	0.948	0.473	0.686
Autism	6	0.300	-0.038	0.639	1.737	0.082	4	0.136	-0.195	0.467	0.806	0.420
**Peer inclusion**												
None	2	0.075	-0.136	0.285	0.695	0.487	1	-0.167	-0.465	0.131	0.447	0.271
Peer involvement	6	0.716	0.123	1.310	2.364	0.018*	4	0.193	-0.137	0.523	1.145	0.252
Peer mediation	2	0.446	-0.212	1.103	1.328	0.184	1	0.570	-0.134	1.273	1.587	0.667
**Facilitator**												
Clinician	4	0.236	-0.198	0.671	1.065	0.287	3	0.013	-0.363	0.388	0.066	0.947
Clinician & Parent	2	0.075	-0.136	0.285	0.695	0.487	1	-0.167	-0.465	0.131	-1.100	0.271
Clinician & Educator	2	0.446	-0.212	1.103	1.328	0.184	1	0.570	-0.134	1.273	1.587	0.113
Educator	2	1.341	0.910	1.773	6.091	<0.001***	1	0.616	0.042	1.190	2.104	0.035*
**Measure informant**												
Child self-report	8	0.671	0.192	1.149	2.745	0.006**	5	0.257	-0.037	0.550	1.711	0.087
Composite	2	0.075	-0.137	0.287	0.695	0.487	1	-0.167	-0.465	0.131	0.447	0.271

Notes

* = *p* < 0.05

** = *p* < 0.01

*** = *p* < 0.001.

#### Between subgroup analyses

The same subgroups used for the within-group analysis were used for the between-group analysis to compare the impact of interventions, but relative to the control comparisons (see Table 4). The intervention effect of the ADHD subgroup of studies was no longer significant once control comparisons (i.e., no intervention or an alternative intervention approach) were accounted for, and the intervention effect for autistic children remained non-significant. The intervention effect for peer involvement was no longer significant. For the facilitator subgrouping, educator-facilitated interventions remained significant and moderate in size (*p* = 0.035, *g* = 0.616, 95% CI = 0.041–1.190). Child self-report outcomes were no longer significant when control comparisons were included.

## Discussion

This study aimed to conduct a systematic review and meta-analysis of interventions for children with a neurodevelopmental disorder known to impact social functioning and to evaluate the impact of those interventions on children’s friendships. To achieve this aim, we analysed the characteristics, methodological quality, and effectiveness of current interventions for improving friendships of children with ADHD, autism, DLD, ID or SPCD. This review identified empirical evidence from 12 studies investigating the effects of interventions on friendships for children with ADHD or autism involving a wide range of intervention approaches. Within-group comparisons yielded overall effects that approached being moderate in size. Between-group comparisons indicated that while large positive effects on friendships were found in some individual studies, overall, the meta-analysis found no significant effect on the friendships of children with a neurodevelopmental disorder relative to control comparisons. However, two interventions, MOSAIC and RR+, found moderate positive effects on children’s friendships relative to their control comparison interventions [51,59]

Educators delivered both MOSAIC and COMET in summer camp classrooms. They used contingency behaviour management strategies to reward children for meeting specific behaviour expectations. MOSAIC included additional strategies to address peers’ perceptions and the inclusion of children with ADHD during summer camp activities. This finding supports the argument that interventions must incorporate techniques to facilitate positive peer perceptions of children with ADHD to have the greatest impact on social functioning [62]. However, results around the impact of COMET and MOSAIC on children’s friendships are potentially inflated relative to other classroom-based interventions in this review due to the context of their implementation. Children participating in MOSAIC and COMET were at reduced odds of having interacted before the intervention period as the interventions were run in a summer camp setting [51]. As a result, the interventions could facilitate the building of positive peer perceptions of children with ADHD from a neutral starting point, which is in stark contrast to other classroom-based interventions reviewed in this study. Interventions delivered in children’s everyday classrooms potentially need to counteract pre-existing negative biases peers may have towards their peers with ADHD, a contextual factor not included in the evaluation of COMET and MOSAIC, which may have attenuated the effect of other class-based interventions relative to these two approaches.

RR+, COMET, and MOSAIC all included intervention components that targeted *child characteristics* that may impact social functioning (i.e., social skills and cognition) among children with a neurodevelopmental disorder, and two included additional elements that focused on peers and their interactions with children with a neurodevelopmental disorder. Interestingly, contrasting techniques were taken to address both elements of social participation. Within RR+, educators were trained to identify autistic children requiring support during break time and scaffold the children’s engagement with peers by supporting social communication, coaching children through challenging social situations with peers and facilitating reciprocal social interactions [59]. As mentioned, COMET and MOSAIC involved contingency behaviour management in encouraging particular social behaviours for children with ADHD [51]. In addition, educators within RR+ worked with typically-developing peers to engage autistic children during recess time, and MOSAIC included educator-modelling, rewards for inclusive behaviour, and drawing positive attention to children with ADHD as additional strategies to increase the likelihood of peer inclusion and to facilitate positive perceptions of children with ADHD by peers [51,59]. While these interventions took contrasting approaches to address child characteristics and peers, collectively, they also support the argument that interventions are likely to have a greater impact on the friendships of children with a neurodevelopmental disorder when they also include strategies that target peers.

The findings that peer inclusion was the most common element among all interventions reviewed add further weight to the argument that peer inclusion is a critical element for intervention approaches to facilitate the development of friendships. Further, intervention effects were greatest when peers were involved, suggesting active involvement of peers within interventions is critical for improving friendships of children with ADHD or autism. Peer involvement as an active ingredient presents many advantages that likely explain its popularity and the increased intervention effects among the interventions reviewed. Involving children’s established social network in an intervention is high in ecological validity, and delivery in schools places the interventions in close proximity to children’s everyday social environment. When features are shared across practice and everyday contexts, the likelihood of generalisation is increased [63]. Peer mediation likely produced the best results because perceptions of peers can also be explicitly addressed, and peers can learn strategies for engaging children with neurodevelopmental differences in social interactions [62].

Subgroup analysis revealed that interventions delivered by educators showed greater improvements in developing friendships compared with other professionals, including those with parental involvement. Furthermore, subgroup analysis showed that including a child self-report measure of friendship is an important consideration for future research.

The broadly non-significant effect of interventions for the between-group comparisons may be explained by the areas of social functioning addressed by the interventions and the outcomes measured. Techniques within most approaches focused on the context of children’s friendships (i.e., child characteristics, peer functioning) rather than on aspects of friendship itself. These interventions potentially had an immediate impact on children’s *peer status*, as improved quality of peer interactions could be associated with raised peer acceptance of children who peers often reject. Positive peer status likely precedes friendship, and studies in this review may have found a greater impact on friendship had longer-term follow-up measures of friendship been taken. Therefore, future evaluations of such interventions should include follow-up friendship measures to provide further insight into the potential downstream effect on friendship. As peer acceptance is also a critical protective factor against bullying and victimisation [64], further investigation of peer status outcomes following interventions is required to understand whether the reviewed interventions do indeed influence this important area of social functioning.

Most studies measured friendships via sociometric nominations. Consequently, findings can only suggest that the intervention approaches reviewed did not have a significant effect on children’s *presence of friendship*. Two studies measured *friendship quality* as an outcome; however, the meta-analysis revealed no significant intervention effect on friendship quality [52,57]. The interventions potentially had a positive effect on *interactions with friends*, given that most approaches focused on children’s interactions with peers; however, no study measured this aspect of social functioning, suggesting a misalignment between the intervention effect and outcomes measured. Elements of social functioning unique to friendship include the *presence of friends*, *characteristics of friends*, *quality of interactions with friends*, and *relationship quality* [65]. While helpful in understanding friendship within the broader context of social functioning, this framework does not shed light on the mechanisms through which children develop and maintain quality friendships. The limited effect of interventions on the *presence of friendship* found in this review provides further support to calls within the literature to compare social skills that are foundational to peer competence and friendship competence to enhance the effectiveness of social skill interventions on friendships [66].

The relationship features that characterise friendships as distinct from peer relationships may also be a more pointed focus for interventions to support friendship development. Several features characterising relationships between friends as distinct from other peer relationships have been identified, namely: proximity, shared activities, similarity, support, assistance, intimacy, trust and reciprocity [67]. Most interventions reviewed supported the proximity and shared activities features of friendship by implementing interventions in schools or classrooms and through peer inclusion. However, most interventions targeted a broad range of social and cognitive skills associated with peer interactions, suggesting further research is required to identify specific skills that are foundational to developing the support, assistance, intimacy, trust and reciprocity features of friendships [66]. The feature of similarity may be better addressed through the targeted selection of peers who are involved in the interventions by consulting with children a priori about peers with whom they have shared interests. The relative importance of these elements of the friend relationship also changes throughout development [68]. For example, proximity and shared activities are important for younger children but become less important compared to intimacy and trust as children become older and into adolescence. Identifying which elements to focus upon through intervention at different developmental stages would be an important area for future research.

Finally, this review only identified studies involving Autistic children or children with ADHD. Interventions involving children with DLD, ID or SPCD are possibly under development but have been evaluated using quasi-experimental, single-group or single-case experimental designs, which were excluded from this review. This finding may result from the increased prevalence of ADHD and autism relative to the other conditions, which are more likely to draw the attention of intervention developers, practitioners and funders. Literature has also identified methodological and practical challenges in researching interventions for individuals with an ID which may reduce the likelihood of RCT-designed studies being implemented and published on this topic [69]. Within the speech pathology literature, a general lack of RCTs for social communication interventions has been recognised [34]. Interventions focusing on conversation skills have overwhelmingly focused on autistic children [70], which may also explain the dearth of intervention studies involving children with DLD, ID or SPCD. In addition, as SPCD is a relatively new diagnosis, the current issues with differential diagnosis and developing interventions take time. Therefore, the lack of RCTs involving children with this diagnosis is perhaps unsurprising. Given the dearth of studies involving these groups, we propose an urgency to develop interventions that positively influence the friendships of children with DLD, ID, or SPCD. In addition, including social functioning and friendship measures in evaluations of effectiveness is essential to develop an evidence-based for implementing interventions that lead to improvements in friendship, which is the true benefit of participation in these interventions for a child.

### Limitations

This study was strengthened by adhering to the PRISMA protocol [41], completing a rigorous search across five databases and implementing a critical appraisal tool to assess methodological quality [43]. However, the decision to only include randomised study designs may have excluded some effective intervention approaches, particularly approaches in the early phases of development that have only been piloted through non-randomised methods. As a result, the study has reviewed effectiveness through studies with ‘gold standard’ methods for establishing effectiveness. While we set out to review interventions involving participants diagnosed with several neurodevelopmental disorders, only interventions involving children with ADHD or on the autism spectrum were found, limiting the generalisability of this study’s findings only to these populations and no other groups. The failsafe N suggests a very low risk of publication bias within the review and meta-analysis; however, meta-analysis results may still need to be interpreted with caution due to the small number of studies we were able to include and because this has led to some small groups within the subgroup analysis. Heterogeneity is present across the participants, interventions, and outcome measures are possible in participant samples; however, this was accounted for within the meta-analysis through random effects modelling.

## Conclusion

Findings from the within-group comparisons are encouraging, providing evidence that individual interventions can positively impact social functioning and foster more meaningful friendships between children with neurodevelopmental disorders and their peers. As a collective, however, studies in this review had a non-significant overall effect on friendships when compared to comparison interventions. This review only identified studies involving Autistic children and children with ADHD. Research using rigorous designs is urgently needed to evaluate the effectiveness of friendship interventions for children with DLD, ID and SPCD.

A wide range of intervention approaches was used; however, effective interventions involved educators in the delivery and targeted child characteristics that may impact social functioning. The active involvement of peers within interventions is a critical element for intervention approaches to facilitate the development of friendships and incorporate techniques to facilitate positive peer perceptions of children and strategies to support peers.

Future research should more comprehensively assess peer functioning, including child self-report measures of friendship, and include follow-up friendship measures to provide further insight into the potential downstream effect on friendship. Finally, future research should identify specific skills foundational to developing the *support*, *assistance*, *intimacy*, *trust and reciprocity* features of friendships and continue to investigate how friendships can be catalysts that enable children to flourish socially and emotionally.

## Supporting information

S1 Checklist PRISMA 2020 main checklist. (PDF)

S1 TableRisk of bias according to the RoB2.*Note*. PEERS = Program for the Education and Enrichment of Relational Skills; RR = Remaking Recess; Low Risk: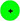; High Risk: 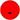; D1 = Randomisation process domain; D2 = Deviations from the intended intervention domain;D3 = Missing outcome data domain; D4 = Measurement of the outcome domain; D5 = Selection of the reported result domain.(DOCX)
