# Supplementary material for: Friendship interventions for children with neurodevelopmental needs: A systematic review and meta-analysis
Source: PLoS One. 2023 Dec 14;18(12):e0295917. doi: 10.1371/journal.pone.0295917 (PMC10721178; doi:10.1371/journal.pone.0295917)
Supplement: S1 Table — Note. PEERS = Program for the Education and Enrichment of Relational Skills; RR = Remaking Recess; Low Risk:; High Risk: ; D1 = Randomisation process domain; D2 = Deviations from the intended intervention domain;D3 = Missing outcome data domain; D4 = Measurement of the outcome domain; D5 = Selection of the reported result domain. (DOCX) [file pone.0295917.s002.docx]

**S1 Table. Risk of Bias according to the RoB2** [47]

| **Study reference** | **Intervention** | **Comparator** | **Outcome** | **D1** | **D2** | **D3** | **D4** | **D5** | **Overall** |
| --- | --- | --- | --- | --- | --- | --- | --- | --- | --- |
| Asmus et al. [57] | Peer Network | Treatment as usual | Social Connections and Relationship Assessment |  |  |  |  |  |  |
| Brock et al. [58] | Peer-Implemented Pivotal Response Training | Treatment as usual | Social Validity Questionnaire |  |  |  |  |  |  |
| Carter et al. [59] | Peer Support Group | Treatment as usual | Social Connections & Relationships Assessment |  |  |  |  |  |  |
| Kasari et al. [74] | ENGAGE | SKILLS | Friendship Survey |  |  |  |  |  |  |
| Kasari et al. [60] | Child-Assisted Approach | Peer-Mediated Approach | Social Network Survey |  |  |  |  |  |  |
| Lerner and Mikami [62] | Sociodramatic Affective Relational Intervention | Skill streaming | Sociometric Nominations |  |  |  |  |  |  |
| Locke et al. [63] | RR | RR with implementation support | Friendship Survey |  |  |  |  |  |  |
| Schohl et al. [64] | PEERS | Waitlist control | Friendship Quality Scale |  |  |  |  |  |  |
| Laugeson et al. [61] | PEERS | Waitlist control | Friendship Quality Scale |  |  |  |  |  |  |
| Mikami et al. [54] | Parental Friendship Coaching | Control (no treatment) | Dishion Social Acceptance Scale |  |  |  |  |  |  |
| Mikami et al. [56] | Parental Friendship Coaching | Coping with ADHD through Relationships and Education | Friendship Quality Scale |  |  |  |  |  |  |
| Mikami et al. [55] | Making Socially Accepting Inclusive Classrooms | Contingency Management Training | Peer Sociometric Nominations |  |  |  |  |  |  |
| Whalen et al. [65] | Medication | Control (no treatment) | Sociometric Assessments |  |  |  |  |  |  |

*Note.* PEERS = Program for the Education and Enrichment of Relational Skills; RR = Remaking Recess; Low Risk: ; High Risk: ; D1 = Randomisation process domain; D2 = Deviations from the intended intervention domain;D3 = Missing outcome data domain; D4 = Measurement of the outcome domain; D5 = Selection of the reported result domain.
